# s-Triazine Derivatives Functionalized with Alkylating 2-Chloroethylamine Fragments as Promising Antimicrobial Agents: Inhibition of Bacterial DNA Gyrases, Molecular Docking Studies, and Antibacterial and Antifungal Activity

**DOI:** 10.3390/ph16091248

**Published:** 2023-09-04

**Authors:** Dawid Maliszewski, Rasime Demirel, Agnieszka Wróbel, Maciej Baradyn, Artur Ratkiewicz, Danuta Drozdowska

**Affiliations:** 1 Department of Organic Chemistry, Medical University of Bialystok, 15-089 Bialystok, Poland; dawid.maliszewski@umb.edu.pl (D.M.); agnieszkawrobel9@gmail.com (A.W.); 2 Department of Biology, Eskisehir Technical University, Eskişehir 26555, Turkey; rasime.demirel@gmail.com; 3 Faculty of Chemistry, University of Bialystok, 15-245 Bialystok, Poland; m.baradyn@uwb.edu.pl (M.B.); artrat@uwb.edu.pl (A.R.)

**Keywords:** triazine scaffold, antibacterial agent, antifungal agent, DNA gyrase, alkylating agents

## Abstract

The spectrum of biological properties of s-triazine derivatives is broad and includes anti-microbial, anti-cancer, and anti-neurodegenerative activities, among others. The s-triazine molecule, due to the possibility of substituting three substituents, offers many opportunities to obtain hybrid compounds with a wide variety of activities. A group of 1,3,5 triazine derivatives containing a dipeptide, 2-ethylpiperazine, and a methoxy group as substituents was screened for their antimicrobial activity. An in vitro study was conducted on pathogenic bacteria (*E. coli*, *S. aureus*, *B. subtilis,* and *M. luteus*), yeasts (*C. albicans*), and filamentous fungi (*A. fumigatus*, *A. flavus*, *F. solani*, and *P. citrinum*) via microdilution in broth, and the results were compared with antibacterial (Streptomycin) and antifungal (Ketoconazole and Nystatin) antibiotics. Several s-triazine analogues have minimal inhibitory concentrations lower than the standard. To confirm the inhibitory potential of the most active compounds against gyrases *E. coli* and *S. aureus*, a bacterial gyrases inhibition assay, and molecular docking studies were performed. The most active s-triazine derivatives contained the -NH-Trp(Boc)-AlaOMe, -NH-Asp(OtBu)-AlaOMe, and -NH-PheOMe moieties in their structures.

## 1. Introduction

A consequence of the overuse and incorrect use of antibiotics and chemotherapeutic agents is the rapid spread of drug-resistant bacteria. This situation is a global problem that affects bacteria both in the community and in the hospital environment. This phenomenon is one of the most serious problems in modern medicine. The search for and synthesis of new compounds that act on bacteria is an urgent necessity in modern science.

This trend is pushing scientists to expand the group of compounds and their applications. In recent decades, due to numerous antimicrobial-resistant infections, forms of modern medicine, like cancer chemotherapy, invasive surgeries, and organ transplantations, can only be performed without risk because of access to effective antimicrobial treatments. Approximately 35,000 deaths/year in the United States and 33,000 deaths/year in Europe are induced by antibiotic-resistant bacteria [1].

Most biologically active compounds are constructed of heterocyclic rings, e.g., pyrrole, imidazole, thiazole, pyrimidine, and triazine. As the latest research has shown, the s-triazine (1,3,5-triazine) scaffold is constantly used to obtain high-potency bioactive derivatives [2]. For example, 1,3,5-triazine derivatives with anticancer activity, with particular emphasis on their inhibition of enzymes involved in the tumorigenesis process, are described in detail [3]. According to ChemSpaider, there are about 114 million chemical compounds in its database, of which about 13,000 are currently used as drugs. This represents about 0.01% of the total population. Among these compounds, there are more than 113,000 s-triazine derivatives, seven of which have been approved as drugs and thirty are in trials, according to DrugBank [4]. Among the known biological properties of s-triazine and its derivatives are antibacterial, fungicidal, antimalarial, anticancer, antiviral, antimicrobial, anti-inflammatory, and antitumor activities.

DNA gyrase is the subject of extensive studies as a target for antimicrobial agents since it is present in bacteria but is lacking in humans [5]. WHO published a list of priority pathogens for which new antibiotics are essential [1,6], including *E. coli* and *S. aureus*, for which priority was established as critical and high, respectively. DNA gyrase is inhibited by fluoroquinolines, aminocoumarin, and NBTI (novel bacterial topoisomerase inhibitors) antibiotics, as well as by simocyclinone, which is an antibiotic comprising an aminocoumarin group and a polyketide group [7]. The fluoroquinolones are examples of broad-spectrum antibacterial drugs targeting DNA gyrase, but because of the increasing bacterial resistance to these agents, there is a need to seek new compounds and new modes of inhibition of this enzyme. Aminocoumarins are competitive inhibitors of DNA gyrase and act by binding to the ATP site within the GyrB subunit [8]. Although simocyclinone has an aminocoumarin moiety in its structure, it does not inhibit the ATPase activity of DNA gyrase but rather binds to the *N*-terminal domain of GyrA and prevents DNA binding [9]. The mechanism of inhibition by fluoroquinolones is that they stabilize the cleaved form of the DNA, preventing the relaxed form of DNA from religating [10]. Lastly, NBTIs have a similar mechanism of action to fluoroquinolones, although they are composed of the “left-hand side”, which intercalates the DNA strands, and the “right-hand side”, which binds into the pocket in the center of the enzyme between two GyrA subunits [11,12].

**Scheme 1:**
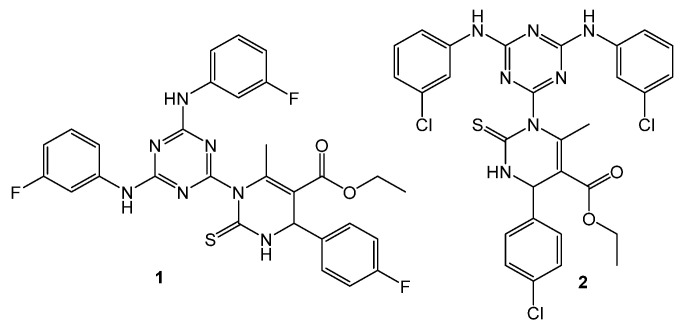
Antibacterial s-triazine derivatives **1** and **2**.

Anup Masih et al. demonstrated antimicrobial potency using an s-triazine derivative combined with a dihydydropyrimidine scaffold (Scheme 1). The antibacterial properties were shown against Gram-positive bacteria, viz. *Staphylococcus aureus*, *Bacillus subtilis*, and *Bacillus cereus*, and three Gram-negative bacterial strains, viz. *Pseudomonas aeruginosa*, *Escherichia coli,* and *Proteus vulgaris*, using the MIC test by **1**. The results were in the range of 3 to 13 µg/mL. The authors explained efficiency as the inhibition of DNA gyrase supercoiling activity (IC_50_ = 3.71 μg/mL). Analog **2** showed great antifungal properties against *Candida albicans*, *Candida glabrata*, *Cryptococcus neoformans*, and *Aspergillus niger* (range of minimum inhibitory concentration from 1.25 to 5.00 μg/mL) [13].

**Scheme 2:**
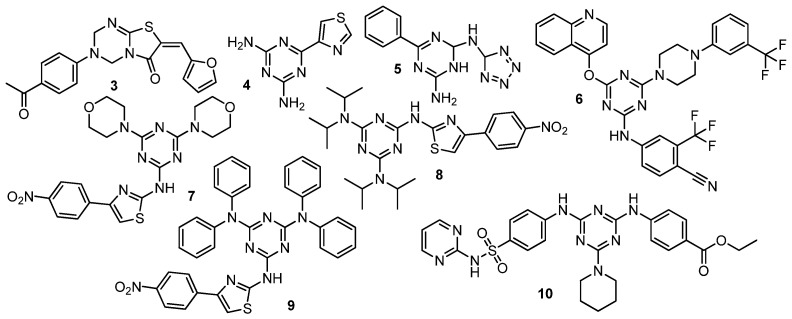
Antibacterial s-triazine derivatives **3**–**10**.

Younis et al. conducted their antibacterial research based on the thiazolo [3,2-a][1,3,5] triazine moiety. The obtained derivative contained substituents on the triazine ring nitrogen, which eliminated the effect of delocalized electrons. Antimicrobial activity was confirmed by studies on *Mycobacterium tuberculosis* lines differing in drug resistance (MIC values for drug-sensitive strain 2.49 μM, multi-drug-resistant strain 9.91 μM, and extensively drug-resistant strain 39.72 μM). Compound **3** (Scheme 2) inhibited 2-trans-enoyl carrier protein reductase (InhA), reaching an IC_50_ value of 3.90 µM. Molecular docking confirmed the activity of this derivative against the tested enzyme. This triazine derivative proved to be not only an antitubercular, but also an antimicrobial potential drug in general, with non-toxic properties against human cells [14].

Patil et al. presented a group of 15 1,3,5-triazine derivatives synthesized in a one-step reaction by mixing 2-cyanoguanidine, potassium hydroxide, and 4-cyanothiazole. Compound **4** (Scheme 2) showed the best antibacterial (*E. coli, K. pneumonia,* and *A. Baumannii*) and antifungal (*C. Neoformans*) properties at a concentration of 32 mg/mL. Molecular modeling showed the generation of hydrogen bonds between **8** and *E. coli* DNA gyrase [15].

The derivative **5** (Scheme 2) demonstrated a strong inhibitory effect on DHFR (IC_50_ = 2.66 μg/mL), DNA gyrase (IC_50_ = 7.54 μg/mL), and *E. coli* TOPO IV (IC_50_ = 1.63 μg/mL). Compound **5** stopped the growth of *E. coli* and *P. aeruginosa* with MIC values of 2.40 ∗ 10^−18^ and 7.12 ∗ 10^−9^ μg/mL, respectively. Additionally, an inhibitory effect was observed on the CYP51 protein (IC_50_ = 7.45 μg/mL), which explains its antifungal activity (*C. albicans* MIC = 1.47 ∗ 10^−8^ μg/mL) [16].

Patel et al. also demonstrated the cytotoxic activity of s-triazine analog **6** (Scheme 2) on bacterial cell lines. The obtained MIC values were at the level of 6.25–12.50 μg/mL, and the inhibition zone was 25–28 mm. The microbiological properties were due to the presence of a strongly electronegative trifluromethyl group [17].

Gahrotri et al. presented derivatives containing a phenylthiazole group. The tests were carried out on Gram-negative (*S. typhi*, *E. coli,* and *K. aerogenes*) and Gram-positive (*B. subtilis*, *B. cereus*, and *S. aureus*) bacterial cell lines. The results showed that compounds **7**–**9** (Scheme 2) achieved excellent MIC values in the range of 4–8 µg/mL [18].

Desei et al. designed and synthesized fifteen s-triazine derivatives containing piperazine and benzenesulfamide moieties. Derivative **10** (Scheme 2) showed significant activity against *P. aeruginosa* (MIC = 25 µg/mL). Research has shown that the electron-withdrawing group at the phenyl group increased the activity of the investigated compounds [19].

The above examples confirm that compounds with an s-triazine ring have great potential in the search for new drugs with antimicrobial and antifungal activities.

This was also confirmed by the study of Liu et al., who, by means of SAR analysis of substituents and biological properties, presented and compared numerous examples of potential antimicrobial compounds [20].

Despite the significant amount of research into s-triazine derivatives in recent years, there is a demand for newer agents with unique, but still effective, mechanisms of action to treat microbial diseases. Alkylating agents, in particular nitrogen-based alkylators, are commonly used, and they act at all phases of the cell cycle. The introduction of an alkylating fragment into the molecule makes the compound more lipophilic, which enables it to penetrate the cell and increase its activity. This applies mainly to cancer cells but can also be effective for bacterial or fungal ones. Nitrogen mustards have still been attracting great interest due to their easy synthesis, lower cost, and high inherent chemical reactivity. Some N-mustard derivatives, including bis(2-bromoethyl)-amine and their azole-substituted compounds such as triazole, imidazole, benzimidazole, etc., showed good biological activity [21].

**Scheme 3:**
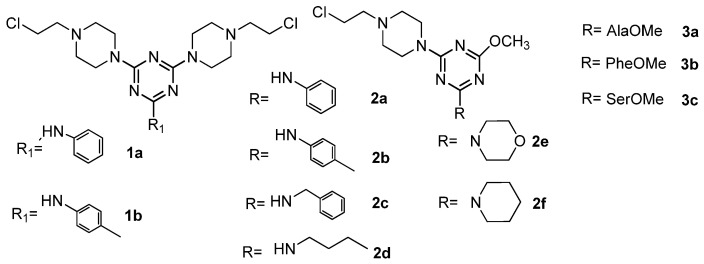
s-Triazine derivatives **1a**,**b**, **2a**–**f**, and **3a**,**b**.

In view of the above, we planned to investigate the antimicrobial potential of the triazine derivatives that we had previously synthesized. The subjects of our interest were 1,3,5-triazine derivatives containing one or two 1-(2-chloroethyl)piperazine groups as well as aromatic, primary, and secondary amines, or amino acids, as substituents (Scheme 3).

**Scheme 4:**
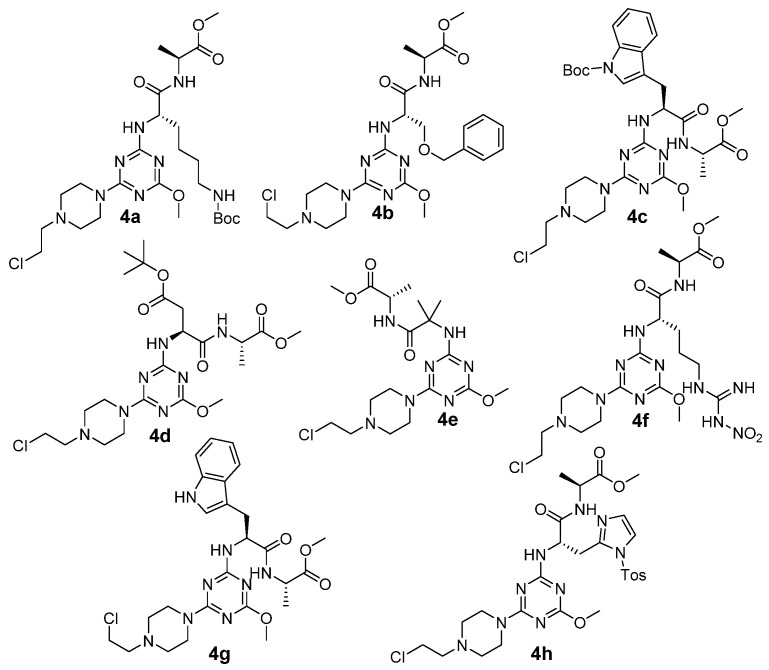
s-Triazine derivatives **4a**–**h**.

These compounds showed antiproliferating activity against MCF-7 and MDA-MB-231 breast cancer cells [22]. The derivatives with amino acids demonstrated promising results, and the derivatives with short peptide chains (Scheme 4) exhibited antineurodegenerative properties [23].

Based on numerous studies reporting the multidirectional activity of s-triazine derivatives [24,25], we wanted to confirm that the compounds we have synthesized and tested for other activities also have potential as antimicrobial agents.

## 2. Results

The structures of all tested compounds are shown in Scheme 3 and Scheme 4. The synthesis as well as the confirmation of identity and purity were performed according to the procedure described earlier [22,23].

The antimicrobial activity of all the compounds was tested in vitro on pathogenic bacteria, yeast, and filamentous fungi using the microbroth dilution method (Table 1) and antibacterial (Streptomycin) and antifungal (Ketoconazole and Nystatin) drugs. Generally, the compounds were more effective against *Candida albicans* than other filamentous fungi and bacteria. In terms of the anticandidal activity of the compounds, they were found to be more successful at lower doses than the tested standard antifungal antibiotics (250 µg/mL). Many of these antifungals have important limitations in their spectrum of activity, pharmacokinetics, and drug–drug interactions, as well as unusual toxicities associated with long-term use [26,27]. For these reasons, the compounds that we have synthesized and examined as new antibiotic candidates are very promising.

In addition to the high anticandidal effects at a low dose, another important finding of our study is that the studied compounds showed broad-spectrum antimicrobial effects. Although some compounds required higher doses than standard antibiotics, compounds **3b** and **4c,d** exhibited the highest inhibitory activity on both bacteria and yeasts such as *S. aureus*, *B. subtilis*, *M. luteus,* and *C. albicans,* with MIC values between 7.81 and 62.50 µg/mL. In particular, **3b** and **4d** showed antibacterial activity at lower doses in *M. luteus* than standard antibiotics (31.25 µg/mL) (Table 1).

Although compounds **1a** and **1b** showed poor antimicrobial activity, the main MIC values of these compounds were found to be 62.50–250 µg/mL for bacteria and 250–500 µg/mL for filamentous fungi. **1b** exhibited anticandidal activity at a lower dose than standard antibiotics for *C. albicans,* with a MIC value of 125 µg/mL (Table 1).

Similarly, we found compound group **2** to be antimicrobial at concentrations between 125 and 500 µg/mL, but **2b**,**d**,**f** showed anticandidal activity at 125 µg/mL MIC, a lower dose than Ketoconazole and Nystatin (Table 1).

Synthetic derivatives from **3a**–**c** and **4c**,**d** proved themselves to be more active against Gram-positive than Gram-negative bacteria. Compound **3b** was the most active (highly effective) against Gram-positive bacilli, especially against *M. luteus*, with an MIC value of 7.81, when the MIC of Streptomycin was 31.25 µg/mL. Derivatives **4c**,**d** had similar activity to **3b** against *S. aureus* and *B. subtilis* but were not more active than Streptomycin. The antibacterial and antifungal activities of compounds **4a**,**b**,**e**–**h** were achieved at concentrations in the 125–500 μg/mL range. We observed high potential for the s-triazine derivative **1b**, containing two nitrogen mustard groups, as well as 4-CH_3_(C_6_H_4_)NH- and analogs with one alkylating chain: **3b** and **4c**,**d**.

All of the compounds showed antifungal activity with an average value of 250 µg/mL, but *C. albicans* turned out to be the most susceptible to compounds **3b** (7.81 µg/mL), **4d** (15.62 µg/mL), and **4c** (62.50 µg/mL).

**Figure 1:**
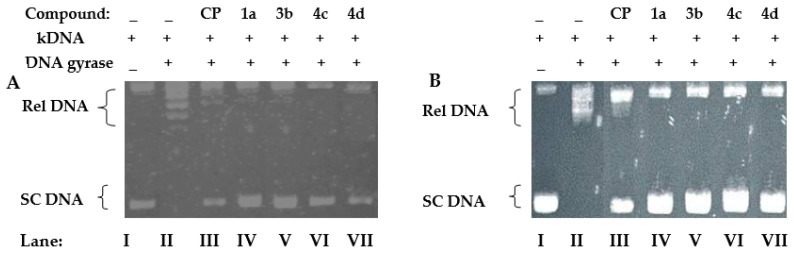
Effects of *S. aureus* (**A**) and *E. coli* (**B**) DNA gyrase on relaxation of kDNA in the presence of compounds **1a, 3b**, and **4c**,**d** (lanes III–VII) at 100 nM. Lane I: kinetoplast DNA (kDNA). Lane II: purified *S. aureus/E. coli* DNA gyrase and kDNA. Lane III: purified *S. aureus/E. coli* DNA gyrase, kDNA, and ciprofloxacin (100 nM). Rel DNA = relaxed forms of DNA; SC = supercoiled DNA.

DNA gyrases continue to be an attractive target and an extremely useful research topic for potential antimicrobial drugs [28]. Ciprofloxacin blocked the activity of *S. aureus* DNA and *E. coli* gyrases [29]. Figure 1 shows the results of electrophoresis analysis of the most active substances, **1a**, **3b**, and **4c**,**d**, after staining with ethidium bromide. At a concentration of 100 nM, we were able to observe all of these s-triazine analogs to have visible inhibiting effects on the ability of *S. aureus* as well as *E. coli* DNA gyrase to transform supercoiled kDNA into several topological forms of relaxed kDNA. It is interesting that 1,3,5-triazine mono [4-(2-chloroethyl)piperazin-1-yl] is a strong alkylating agent with the most nucleophilic functional groups, which is typical for nucleic acids and proteins.

**Figure 2:**
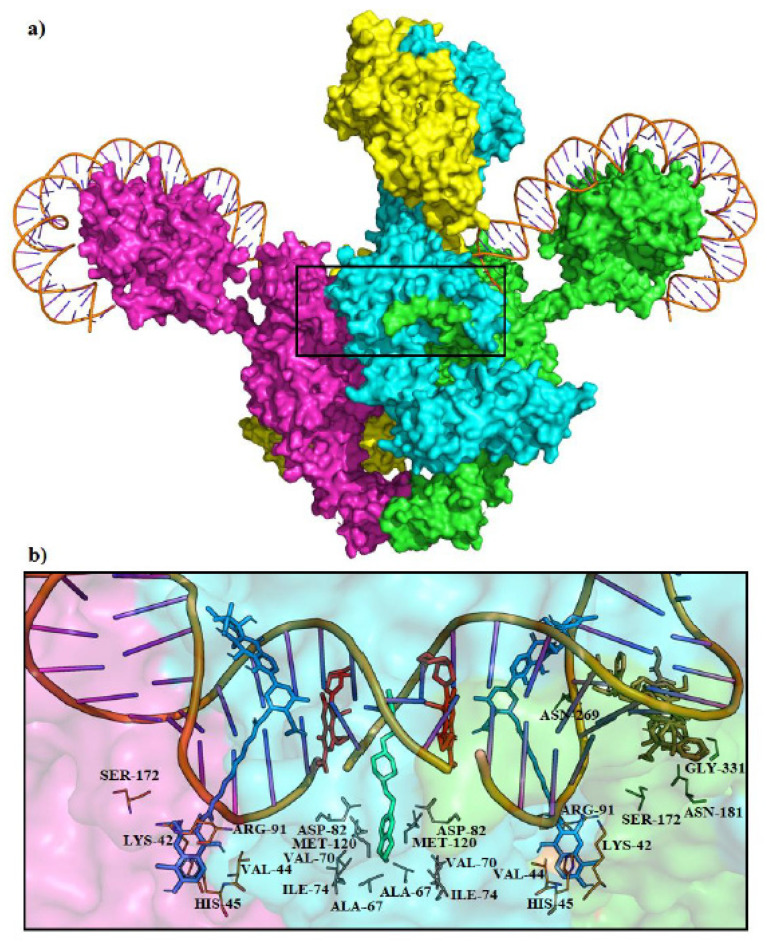
Structure of *E. coli* DNA gyrase bound to a DNA duplex (PDB: 6RKW). (**a**) The surface of the protein is colored according to the subunit of the enzyme, where magenta and green are GyrA subunits and blue and yellow are GyrB subunits. The potential inhibitor binding area is marked with a black rectangle. (**b**) Close-up of the potential inhibitor binding area with DNA duplex and residues involved in binding inhibitors of DNA gyrase. Other inhibitors are presented for comparison: red—moxifloxacin (PDB: 4Z2C); blue—simocyclinone D8 (PDB: 2Y3P); and aquamarine—gepotidacin (PDB: 6RKW). Inhibitors **3b** and **4c**,**d**, are superimposed and colored brown. Residues that belong to the simocyclinone binding site are orange, residues in the NBTI binding site are gray, and residues involved in binding our inhibitors are green.

The mechanism of action of the tested 1,3,5-triazine derivatives needs further study. We have, therefore, planned a computer-based study and begun our molecular docking studies by investigating potential binding sites for our inhibitors. Since Streptomycin was used as a control antibiotic to examine the antibacterial activity of our molecules, it was also included in the molecular docking study. As mentioned in the methods section, we performed a scan of the whole DNA-binding/cleavage domain to look for potential binding sites. The theoretical experiment showed that our inhibitors do not fit into the typical binding sites reported in the literature [7,10,11,12,20,30]. Instead, the binding modes from all investigated sites during the scan indicated that the tested molecules have a strong preference for binding with DNA. It was found that all inhibitors showed the highest affinity for the same site, located at the minor groove of DNA next to the simocyclinone binding site (Figure 2). This was the case for both *E. coli* and *S. aureus* DNA gyrases. We have also observed, as it is with other inhibitors (Figure 2), that our derivatives bind symmetrically into the suggested binding site in each GyrA subunit, showing the same binding energy and binding modes. Therefore, we only present results for one of the two identical binding sites.

To fully explore the activity of our inhibitors, we conducted a molecular docking study at the ATP/aminocoumarin binding site within the GyrB subunit of DNA gyrase. In the case of *E. coli*, the complete enzyme structure was available (PDB: 6RKW), but for *S. aureus,* the *N*-terminal domain of DNA gyrase GyrB (24 kDa) was obtained from the Protein Data Bank (PDB: 4URO) [31]. The grid box with dimensions 20 × 20 × 20 Å was set at the ATP binding pocket. The resulting ATP side binding energies were: −7.8 kcal/mol for **3b**, −7.7 kcal/mol for **4d**, and −7.4 kcal/mol for **4c** in the case of *E. coli,* and −7.3 kcal/mol for **3b**, −6.0 kcal/mol for **4d**, and −7.1 kcal/mol for **4c** in the case of *S. aureus*. However, they were significantly lower when compared to the binding energies at the DNA-binding/cleavage domain (Table 2). For that reason, we have not reported details about the binding modes at the ATP binding site.

**Figure 3:**
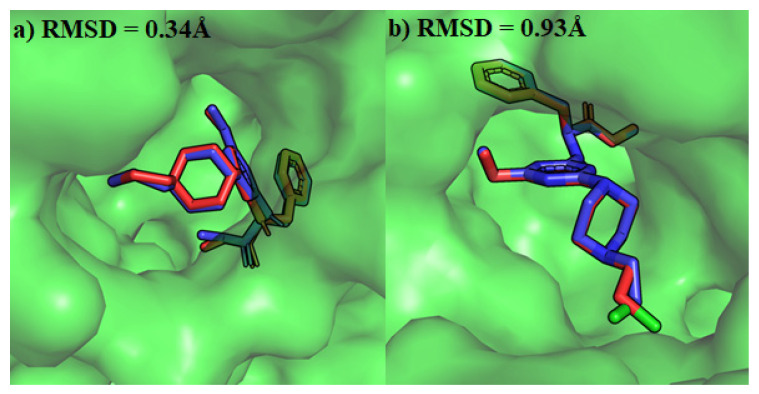
Superimposed binding modes of **3b** estimated by AutoDock Vina (red) and the binding modes of 3**b** from the Blind Docking Server (blue) within the putative binding sites of (**a**) *E. coli* (PDB: 6RKW) and (**b**) *S. aureus* (PDB: 7MVS).

Calculations were carried out with the Blind Docking Server [32], available at http://bio-hpc.eu/software/blind-docking-server/ (accessed on 22 August 2022), to find the binding site of our inhibitors for both bacterial DNA gyrases. This tool performs an exhaustive series of docking calculations across the whole protein surface in order to find the spots with the best binding affinities. However, only **3b** could be used as the molecule for the Blind Docking calculation, since it only allows for small ligands with up to 12 degrees of freedom. The results showed that this inhibitor had the highest affinity at the same location in both enzymes as in our study (Figure 3), which further validates our assumption about this binding site. The binding energies from the Blind Docking Server were −9.3 kcal/mol for *E. coli* and −9.4 kcal/mol for *S. aureus*. These values are comparable to those in our study, which were estimated by AutoDock Vina: −8.8 kcal/mol for *E. coli* and −9.2 kcal/mol for *S. aureus*.

Binding energies, inhibition constants, and ligand efficiencies are presented in Table 2. It can be seen that the best binding energy in the cases of both *E. coli* and *S. aureus* was inhibitor **4c**. However, the value of ligand efficiency shows that the most promising derivative was **3b**, followed by **4c**,**d**. Ligand efficiency is a value that expresses the binding energy of a compound normalized by the compound’s size and is an important property to consider when assessing the quality of binding modes. Larger compounds tend to show greater binding energy due to the larger number of interactions they form in molecular docking experiments, but they may not necessarily be the most efficient inhibitors [7].

**Figure 4:**
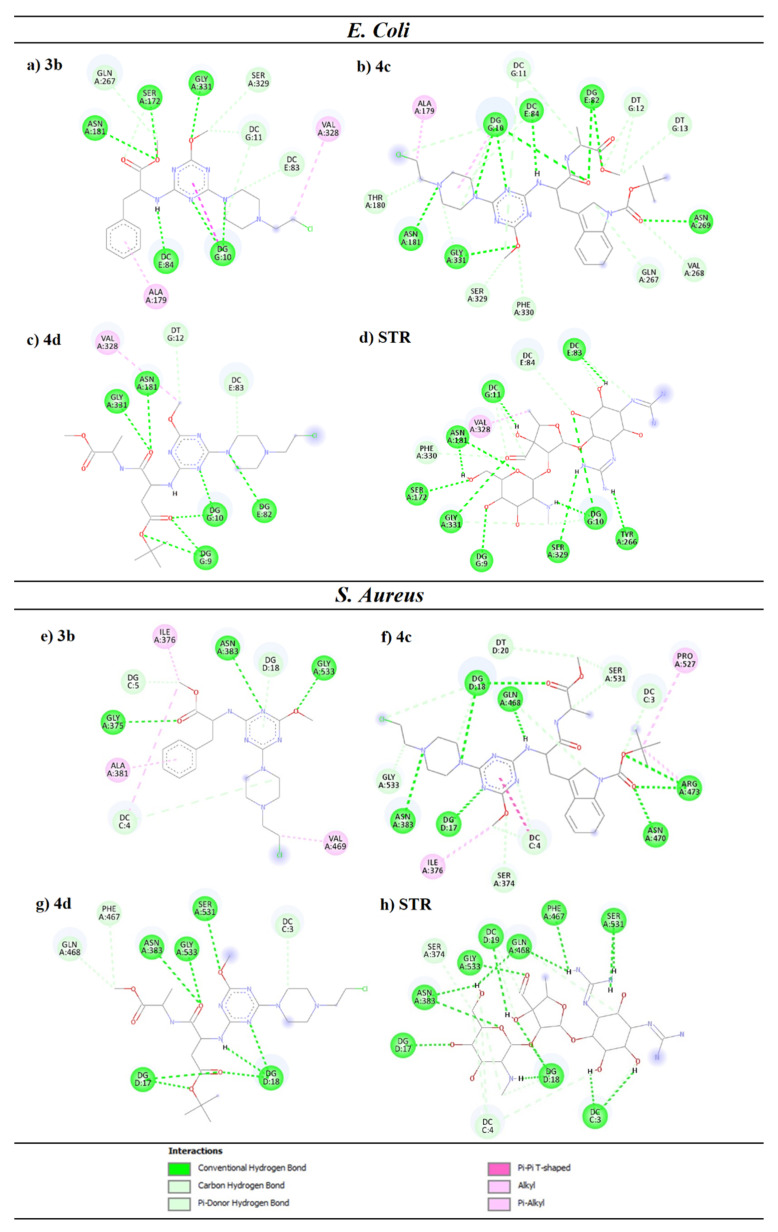
Interactions between inhibitors from this study and residues or nucleotides from molecular docking studies. Subfigures (**a**–**d**) refer to ligands **3b** and **4c**,**d** and Streptomycin (**STR**) bound to the *E. coli* DNA gyrase (PDB: 6RKW). Subfigures (**e**–**h**) refer to ligands **3b**, **4c**,**d**, and **STR** bound to the *E. coli* DNA gyrase (PDB: 7MVS).

As with binding energy, the lower the value of ligand efficiency, the more affinity it shows towards the receptor. Using the BIOVIA Discovery Studio software, we managed to find interactions involved in forming DNA gyrase–ligand complexes. As can be seen in Figure 4, mainly hydrogen bonds (H-bonds) were formed, although a few hydrophobic interactions as well as π–π interactions were observed. In most cases, our inhibitors had a tendency to bind with nucleotides in the minor-groove of DNA, but interactions with residues in the GyrA subunit were also present. This was to be expected since our inhibitors were analogous to minor-groove binding agents such as netropsin or distamycin. The details of the H-bonds formed between our derivatives and receptors are presented in Table 3.

**Figure 5:**
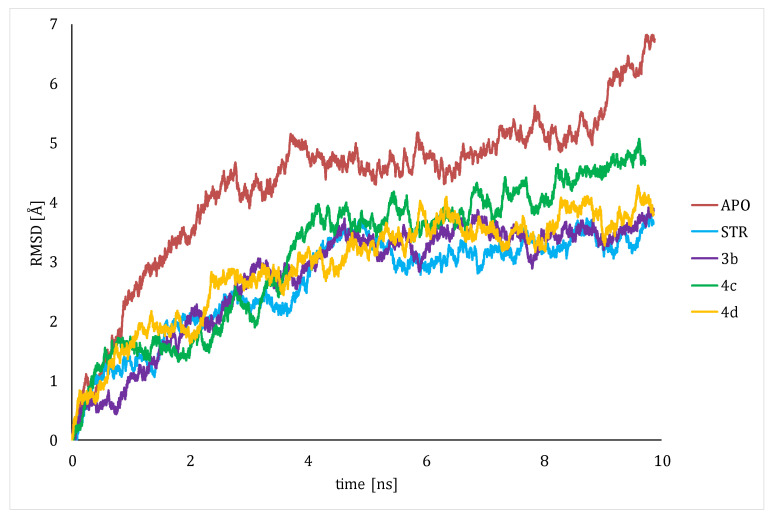
RMSD analysis for the unliganded enzyme 6RKW (APO) and its complexes with derivatives **3b**, **4c**,**d**, and **STR**.

To gain deeper insight into the protein and docked complexes under physiological conditions, molecular dynamics simulations were performed on the docking of the most promising drug candidates, namely **3b**, and **4c**,**d**, to the unliganded (apoenzyme) form of the *E. coli* gyrase (6RKW). For the sake of comparison, the docking of Streptomycin to the same protein was also modeled. The RMSD—as an abbreviation for root mean square deviation—means the Euclidian distance of atoms within a simulation. RMSD analysis of the alpha carbons of all systems allows for estimating their stability during simulation. The results for the systems investigated here are plotted in Figure 5. It is seen that all tested ligands, including Streptomycin, have a tendency to lower this value vs. apoenzyme during the simulation, thus showing a stabilizing effect. All curves are essentially similar, so it is difficult to distinguish the ligand that lowers the RMSD the most. Only the influence of derivative **4c** on protein stabilization seems to be somewhat less significant. This conclusion is also supported by the average RMSD values over the entire 10 ns launch, which are (Å): 3.83 (**APO**), 2.95 (**STR**), 2.75 (**3b**), 3.13 (**4c**), and 2.69 (**4d**).

The most important conclusion of docking is the localization and accurate characterization of the interaction of ligands with the active center of the enzyme. Since hydrogen bonds are the main factor stabilizing the ligand position, investigation of the time evolution of their creation and disappearance provides fundamental data on the stability of the forming complexes. This is performed by noting the number of hydrogen bonds between receptor and ligand during the MD simulation. Results are given in Table 4. A closer look at this table reveals the differences between the docking and MD results. In fact, some of the most common contacts in MD simulations are not present in Figure 4, illustrating the exact position of ligands docked in the enzyme, which is the starting point of MD analysis. This is not surprising since docking is performed on a static crystallographic structure that fluctuates during the MD run. For derivative **3b**, the most common bond with LYS-76 appears a few picoseconds from the start of the simulation, when the ligand approaches the terminal amino group of the lysine, with which it remains in contact for more than 3/4 of the simulation. The same is true for the **4d** derivative, which is initially about 3.5 Å away from arginine-272 (ARG-272), a distance that is a bit too far for hydrogen bond formation. As the modeling proceeds, **4d** approaches the amino acid, forming a bond between the amino group of ARG-272 and the piperazine ring of the ligand. A common element in all the dominant H-contacts is GLY-331, which appears in all ligands tested both at and after docking. However, in spite of similar docking poses, there is a noticeable difference between the dynamic interactions of our proposed ligands and Streptomycin. While derivatives **3b** and **4c**,**d** are mainly in contact with the enzyme’s amino acids, contacts with DNA strands dominate for Streptomycin, occurring for more than 50% of the simulation time. This suggests a different mechanism of inhibition. While our ligands bind mainly to chain A of enzymes, Streptomycin inhibits DNA division by locking the DNA itself rather than the enzyme cutting it.

The next analysis consists of determining, using QSAR methodology, the values of certain pharmacokinetic parameters, the compilation of which will be the next step towards clarifying the differences in bioactivity of our proposed derivatives in relation to Streptomycin (Table 1). In particular, the parameters describing the potential toxicity of the compound and those possibly related to its absorption into the body were determined. The results are summarized in Table 5. The first observation that arises is the difference in the estimated values of logP, which is a measure of the lipophilicity of a molecule. This parameter is several orders of magnitude lower for Streptomycin than for the new derivatives. In addition, Streptomycin is not absorbed in the intestines and is not a P-glycoprotein inhibitor, which is also important in the context of drug absorption in the body. Another parameter indicating poorer absorption is Plasma Protein Binding, which is significantly lower for Streptomycin than for the other ligands. Therefore, it can be concluded that the calculated parameters indicate better absorption of the new derivatives than Streptomycin. This is also related to the number of hydrogen bonds formed by the molecule in question, which is clearly higher for Streptomycin. On the other hand, the results clearly indicate that STR is more toxic than each of the other molecules. For these reasons (better bioavailability and lower toxicity), it can be concluded that our ligands are worthy drug candidates for consideration. Of these, the **b3** derivative appears to have the fewest side effects.

## 3. Discussion

Tests carried out on fungi and bacteria showed the antimicrobial properties of the mentioned compounds, in particular compounds **4c**,**d**. Studies on *E. coli* gyrase and *S. aureus* revealed the inhibitory potential of topoisomerase II. The theoretical experiment showed that inhibitors do not fit into the typical binding sites reported in the literature [7,10,11,12,29]. Instead, the binding modes from all investigated sites during the scan indicated that the tested molecules have a strong preference for binding with DNA. It was found that all inhibitors showed the highest affinity for the same site, located at the minor groove of DNA next to the simocyclinone binding site (Figure 2). This was the case for both *E. coli* and *S. aureus* DNA gyrases. Theoretical calculations allowed us to find the active site on the enzyme’s topography and confirm the inhibitory nature of the compounds. Molecular docking studies have shown that our inhibitors have a high affinity for the DNA gyrase enzyme in both *E. coli* and *S. aureus*. Therefore, we suggest a new binding site for our molecules involving residues Ser-172, Asn-181, Asn-269, and Gly-331 in the case of *E. coli* and Gly-375, Asn-383, Gln-468, Asn-470, Arg-473, Ser-531, and Gly-533 in the case of *S. aureus* (Figure 4 and Table 4). However, further experimental studies are required in order to unambiguously confirm our assumption about this binding site. Binding energies, inhibition constants, and ligand efficiencies are presented in Table 3. It can be seen that the best binding energy in the cases of both *E. coli* and *S. aureus* was inhibitor **4c**. However, the value of ligand efficiency shows that the most promising derivative was **3b**, followed by **4c**,**d**. As with the binding energy, the lower the ligand’s efficiency value, the greater its affinity for the receptor. We can see from Table 2 and Table 3 that there was a correlation between the experimental values of MIC and the ligand efficiency from theoretical calculations. This affinity is better reflected when comparing the values of ligand efficiency, taking into consideration the molecule size. After selecting the best docking derivatives, a deeper evaluation of their interaction with the receptor was carried out through MD simulations and quantitative structure-activity relationship (QSAR) modeling. The results showed significant differences between the proposed derivatives and Streptomycin, both in mechanism of activity and toxicity. While the former method does not identify a clear favorite, it does highlight significant differences in the mechanisms of action of proposed derivatives compared to a well-known drug that is long established on the market. QSAR analysis indicates the low toxicity of the new derivatives and their good bioavailability. Here, derivative **3b** performs best. The theoretical and experimental results are in great agreement and show that the most potent inhibitors in our study were **3b** and **4c**,**d**.

Due to the increasing drug resistance of microorganisms, it is necessary to develop therapeutic methods based on chemotherapy, and topoisomerase II is an important target. The triazine scaffold is very well suited for development and modification in order to obtain more active compounds to overcome the drug resistance of microbes. The obtained results suggest that future studies should expand the group of tested microorganisms as prospective targets. In the next stage of testing, it is important to include controls with non-susceptible or resistant bacteria. It is also important to investigate the activity of our compounds against strains of resistant bacteria, as activity and resistance may be due to the structure of the compounds and also depend on the resistance mechanisms of the bacteria, which may interfere with the observed results.

It is also important to carry out research on improving the framework of the derivatives to improve their antimicrobial activity. Other amino acids in compound **3b,** as well as other dipeptides in the **4c**,**d** analogues, can be tested as activating elements. The most promising seem to be glycine, arginine, proline, histidine, and tryptophan. These amino acids, as well as their combinations, appear to be worth introducing into the molecule as they play significant roles in the antimicrobial activity of the peptides [33].

## 4. Materials and Methods

### 4.1. Study on Selected Cell Strains of Fungi and Bacteria

The antimicrobial activities of compounds were assessed by using MIC according to CLSI M27-A2 [34] for bacteria and CLSI M07-A10 [35] and CLSI M38-A20 for yeasts and filamentous fungi, respectively, with some modifications. The tested microorganisms were some bacteria, yeasts, and filamentous fungi, including *Bacillus subtilis* (NRS-744), *Escherichia coli* (ATCC-25922), *Staphylococcus aureus* (NRRL B-767), *Candida albicans* (ATCC-90028), *Aspergillus flavus* (NRRL-980), *Aspergillus fumigatus* (NRRL 163), *Fusarium solani* (NRRL-13414), and *Penicillium citrinum* (NRRL 1841), respectively. A microbroth dilution susceptibility assay was used for the antimicrobial evaluation of the compounds. Stock solutions of the samples were prepared in dimethyl sulfoxide (DMSO). A dilution series using sterile distilled water was prepared from 4 mg/mL to 0.0039 mg/mL in micro-test tubes, which were transferred to 96-well microtiter plates. Overnight-grown bacteria and *C. albicans* suspensions in double-strength Mueller–Hinton broth were standardized to 10^8^ CFU/mL by using McFarland No. 0.5 standard solutions, and 10^5^ cell/mL spore/mL suspension in 1% Tween 80 was used for yeasts in double-strength MHB and filamentous fungi in double-strength potato dextrose broth, respectively. Then, 100 µL of each microorganism suspension was added to the wells. The last well chain without an added microorganism was used as the negative control. Sterile distilled water and the medium served as the positive growth control. After incubation for 24 h at 37 °C for bacteria and yeasts and for 25 °C at 72 h for filamentous fungi, the minimum inhibitory concentration was detected by spraying 0.5% TTC (triphenyl tetrazolium chloride, Merck) aqueous solution for bacteria and yeasts and by investigation of mycelia growing under a stereomicroscope for filamentous fungi. Streptomycin, Nystatin, and Ketoconazole were used as control antibiotic agents.

### 4.2. Relaxation Assay of Escherichia coli and Staphylococcus aureus Gyrases

Kinetoplast DNA (kDNA) (0.20 µg) was incubated with 1 U of *S. aureus* gyrase (reaction mixtures for the gyrase supercoiling assays contained: 35 mM Tris-HCl (pH 7.5), 24 mM KCl, 700 mM K-Glu, 4 mM MgCl_2_, 2 mM DTT, 1.8 mM spermidine, 1 mM ATP, 6.5% (*w*/*v*) glycerol, and 0.1 mg/mL albumin) or 1 U of *E. coli* gyrase (reaction mixtures for the gyrase supercoiling assays contained: 40 mM Tris pH 7.5, 6 mM MgCl_2_, 10 mM DTT, 100 mM potassium glutamate, 50 mg/mL acetylated BSA, and 1 mM ATP) in the absence or presence of varying concentrations of the test compounds, with ciprofloxacin as positive control (100 nM). Enzyme activity was detected by incubation for 45 min at 37 °C in a total reaction volume of 10 μL, and the reaction was terminated by the addition of 2 µL of 10% SDS. The reaction mixture was subjected to electrophoresis (2 h, 110 V) through a 1.0% agarose gel in TBE buffer (90 mM Tris-borate and 2 mM EDTA). The gels were stained for 30 min with EtBr solution (0.5 µg/mL). The DNA was visualized using a 312 nm wavelength transilluminator and photographed under UV light. For the quantitative determination of topoisomerase activity, the area representing supercoiled DNA migrating as a single band at the bottom of the gel was measured using the InGenius gel documentation and analysis system (TK Biotech, Warszawa, Poland). The concentrations of the compounds that converted 50% of the supercoiled DNA (IC_50_ values) were determined by averaging the data from at least three experiments.

### 4.3. Molecular Docking of Escherichia coli and Staphylococcus aureus Gyrases

There are a number of available bacterial DNA gyrase structures that can be found in the Protein Data Bank. Recently, the complete structure of *E. coli* DNA gyrase was elucidated using the cryo-EM technique [11], which provided a solid foundation for drug discovery. The structures of *E. coli* (PDB: 6RKW) as well as *S. aureus* (PDB: 7MVS) [31] DNA gyrases were obtained from the Protein Data Bank. In each case, the enzyme was prepared for calculations by removing water molecules and co-crystallized ligands, as well as adding polar hydrogen atoms. The software used for molecular docking was AutoDock Vina v.1.2.0 [32]. The BIOVIA Discovery Studio software was used to search for residues and nucleotides involved in the binding of the studied ligands. To validate our method, we used the Blind Docking Server [36], available at: http://bio-hpc.eu/software/blind-docking-server/ (accessed on 22 August 2022), which performs an exhaustive series of docking calculations across the whole protein surface.

Binding energies, inhibition constants, and ligand efficiencies were calculated for each ligand enzyme complex. Binding energy is a value that represents the affinity of a ligand binding to the receptor and is calculated using the Autodock Vina scoring function. The inhibition constant (K_I_) was calculated from the binding energy (ΔG) using the formula Ki = exp(ΔG/RT), where R is the universal gas constant (1.985 × 10^−3^ kcal mol^−1^ K^−1^) and T is 298.15 K. Ligand efficiency is the binding energy (ΔG) divided by the number of non-hydrogen atoms [37].

### 4.4. Molecular Dynamics

Simulations were conducted with the NAMD 2.14 program [38] and the CHARMM22 force field with CMAP correction to proteins [39,40]. The individual parameter files (prm) for ligands were prepared with the Ligand Reader and Modeler from the CHARMM-GUI web environment [41,42]. Due to the huge size of the system (30244 non-hydrogen atoms + ligand), simulations with explicit solvent modeling would have been too time-consuming, so a generalized implicit Born solvent model with a default (i.e., 12Å) cutoff used to determine the Born radius of each atom was utilized. The systems studied were initially minimized for 50,000 steps, gradually heated from 0 to 310 K in 2 K increments, and equilibrated with an additional 500,000 steps of 2 fs. Then, for consequent trajectory analysis, the production runs were performed for 10 ns with a time step of 2 fs and frames saved every 1000 steps. A constant temperature (T = 310 K) was enforced by Langevin dynamics with a damping coefficient of 1/5 ps.

### 4.5. Pharmacokinetic Properties

Toxicity and some pharmacokinetic parameters related to intracellular drug absorption have been predicted using admet SAR online [43]. By eliminating species with undesirable properties in the early stages of drug discovery, pharmacokinetic studies and data analysis can help select the most promising derivative.

## 5. Conclusions

Among nineteen 1,3,5-triazine derivatives subjected to microbiological tests, the compounds **3b** and **4c**,**d** showed the strongest bactericidal activity. Their structures contain methoxyl, 2-chloroethylpiperazine, and, respectively, -NH-Trp(Boc)-AlaOMe, -NH-Asp(tBu)-AlaOMe, and -NH-PheOMe moieties. These compounds showed more potent activity against fungi, but the results were weaker than those observed for bacteria. Aromatic substituents and aliphatic carbon chains reduced the microbial activity of the s-triazine derivatives.

Investigating the mechanism of action of these compounds, we found that the tested compounds inhibited the bacterial gyrases of *E. coli* and *S. aureus*. They blocked the relaxation process of DNA and stopped cell growth. Molecular docking confirmed these results and showed a strong effect on the GyrA subunit at the site of the DNA minor groove. As proven in the molecular dynamics simulations, incorporation of the ligand blocked the proper complexation of the gyrase and DNA helix, which may have resulted in incorrect operation of the enzyme. QSAR studies have shown that the suggested best derivatives are safe to use. Further research into the effect of the presence of a 2 chloroethylpiperazine substituent, as well as dipeptide fragments built from other amino acids, offers the prospect of obtaining derivatives with high biological activity.

## Figures and Tables

**Table 1:** Antibacterial and antifungal activities of various compounds (μg/mL).

Antibacterial Activities					Antifungal Activities				
Compound	A	B	C	D	E	F	G	H	I
**1a**	250	250	250	125	250	250	500	250	250
**1b**	250	250	125	62.50	250	250	500	250	**125**
**2a**	250	250	250	250	250	250	500	250	250
**2b**	250	250	250	125	250	250	250	250	**125**
**2c**	250	250	250	250	250	250	250	250	250
**2d**	250	125	125	125	250	250	500	250	**125**
**2e**	500	500	500	500	250	250	250	250	500
**2f**	250	250	250	125	250	250	500	250	**125**
**3a**	500	500	250	250	250	250	500	250	250
**3b**	500	**62.50**	**31.25**	**7.81**	250	250	500	250	**7.81**
**3c**	250	250	250	250	250	250	500	250	250
**4a**	250	500	250	250	250	250	500	250	250
**4b**	250	250	250	250	250	250	500	250	**125**
**4c**	125	125	**62.50**	**62.50**	250	250	500	250	**62.50**
**4d**	125	**62.50**	**31.25**	**15.62**	250	250	500	250	**15.62**
**4e**	250	250	250	250	250	250	500	250	250
**4f**	250	500	500	250	250	500	250	250	**125**
**4g**	250	500	250	250	250	250	500	250	250
**4h**	250	500	500	250	500	250	250	250	250
Streptomycin	15.62	31.25	7.81	31.25	-	-	-	-	-
Ketoconazole	-	-	-	-	62.50	15.62	62.52	31.25	250
Nystatin					3.90	3.90	3.90	1.95	250

A: *E. coli* (ATCC-25922), B: *S. aureus* (NRRL B-767), C: *B. subtilis* (NRS-744), D: *M. luteus* (NRRL B-4375), E: *A. fumigatus* (NRRL 163), F: *A. flavus* (NRRL 980), G: *F. solani* (NRRL 13414), H: *P. citrinum* (NRRL 1841), and I: *C. albicans* (ATCC 90028). The best results are in bold.

**Table 2:** Binding energies, inhibition constants, and ligand efficiencies of the tested ligand–enzyme complexes. Inhibition constants and ligand efficiencies were calculated based on the binding energies from the AutoDock Vina scoring function.

Gyrase	Compound	Binding Energy (kcal/mol)	Inhibition Constant (μM)	Ligand Efficiency
*E. coli*	**3b**	−8.8	0.354	−0.29
	**4d**	−8.8	0.354	−0.24
	**4c**	−10.0	0.047	−0.22
*S. aureus*	**3b**	−9.2	0.180	−0.31
	**4d**	−8.6	0.496	−0.24
	**4c**	−10.2	0.033	−0.23

**Table 3:** Residues and nucleotides involved in binding of **3b** and **4c,d,** and Streptomycin (**STR**) with the lengths of H-bonds.

E. coli						S. aureus					
No.	Residue	H-Bond Length [Å]	No.	Residue	H-Bond Length [Å]	No.	Residue	H-Bond Length [Å]	No.	Residue	H-Bond Length [Å]
**3b**	DG10	2.33	**4c**	DG82	2.62	**3b**	Gly-375	2.88	**4c**	Asn-470	2.32
	DG10	2.73		DG82	2.97		Asn-383	2.73		Arg-473	2.8
	DC84	2.25		DC84	2.28		Gly-533	2.21		Arg-473	2.9
	Ser-172	2.68		Asn-181	2.47	**4d**	DG17	2.33	**STR**	DC3	1.77
	Asn-181	2.77		Asn-269	2.88		DG17	3.07		DC3	2.46
	Gly-331	2.41		Gly-331	2.57		DG18	2.52		DG17	2.97
**4d**	DG9	2.38	**STR**	DG9	2.35		DG18	2.7		DG18	2.42
	DG9	2.34		DG10	2.34		DG18	2.72		DG18	2.11
	DG10	3		DG10	3.1		Asn-383	2.75		DC19	2.63
	DG10	2.42		DC11	2.08		Gly-533	2.12		Asn-383	2.84
	DG82	2.54		DC83	3.06		Ser-531	2.51		Asn-383	2.94
	Asn-181	2.46		Ser-172	2.54	**4c**	DG17	3.01		Phe-467	2.42
	Gly-331	2.04		Asn-181	2.83		DG18	2.23		Gln-468	2.57
**4c**	DG10	2.63		Tyr-266	2.41		DG18	2.89		Gln-468	2.84
	DG10	2.76		Ser-329	2.84		Asn-383	2.78		Ser-531	2.33
	DG10	2.84		Gly-331	2.93		Gln-468	1.91		Gly-533	2.25

**Table 4:** List of H-bonds between the tested molecules and residues in the active site of gyrase *E. coli*. The type of interaction indicates whether a ligand is a donor (D) or acceptor (A). Only residues with an occupancy higher than 5% are listed.

Ligand	Donor	Acceptor	Occupancy	Ligand	Donor	Acceptor	Occupancy
**3b**	LYS-276	3b	77.20%	**4d**	ARG-272	4d	87.10%
	ASN-269	3b	19.71%		GLY-331	4d	49.20%
	GLY-331	3b	17.49%		DG-10	4d	7.60%
	DG-82	3b	15.94%		DG-82	4d	5.20%
	3b	SER-329	11.21%	STR	STR	DC-84	51.68%
	DG-10	3b	6.86%		STR	DG-85	47.72%
	DT-13	3b	5.31%		GLY-331	STR	22.78%
**4c**	GLY-331	4c	34.90%		STR	DC-83	19.66%
	SER-172	4c	32.20%		DG-82	STR	16.79%
	DG-82	4c	19.30%		ASN-181	STR	9.35%
	4c	DC-83	14.50%		STR	SER-329	9.35%
	ASN-181	4c	13.20%		DG-10	STR	8.27%
	GLY-173	4c	12.10%		STR	DC-11	7.31%
	DG-10	4c	9.80%		ARG-237	STR	6.95%
	ARG-272	4c	5.60%		STR	GLN-325	6.83%
					ASN-269	STR	6.24%
					DG-9	STR	5.52%
					STR	DT-12	5.04%

**Table 5:** Pharmacokinetic parameters of the best-selected derivatives and Streptomycin. D symbolizes H bond donor, A—acceptor, y—yes, n—no.

Ligand	Hbond D/A	AlogP	Acute Oral Toxicity Log (1/(mol/kg))	Carcinogenicity/Hepa/Skin/Respiratory/Mitochondrial/Nephro-Toxicity	Plasma Protein Binding (100%)	Blood-Brain Barrier	Human Intestinal Absorption	Ames Mutagenesis	P-Glycoprotein Inhibition/Substrate
**3b**	1/9	1.61	2.304	n/n/n/y/y/n	0.775	y	y	n	y/y
**4c**	2/11	2.85	2.528	n/n/n/y/y/n	0.761	y	y	n	y/y
**4d**	2/12	0.6	2.146	n/y/n/y/y/y	0.405	y	y	y	y/y
STR	12/15	−8.16	2.123	n/y/y/y/y/y	0.177	n	n	y	n/n

## Data Availability

Data is contained within the article.

## Abbreviations

Aib—alpha-aminoisobutyric acid; Ala-OMe—L-alanine methyl ester; Arg (NO_2_)—nitro-L-arginine; Asp (OtBu)—L-Aspartic Acid 4-tert-Butyl Ester; Boc—tert-Butoxycarbonyl protection group; CYP51—Candida 14 α-demethylase enzyme; DHFR—Dihydrofolate Reductase; DTT—Ditiotreitol; GyrA, GyrB—subunits of DNA gyrase; His (Tos)—tosyl-L-histidine; InhA—2-trans-enoyl-acyl carrier protein reductase; kDNA—kinetoplast DNA; Lys (Boc)—Boc-L-lysine; MDA-MB-231—estrogen MIC—minimum inhibitory concentration; NBTI—Novel Bacterial Topoisomerase Inhibitors; PDB—Protein Data Bank; Rel DNA—relaxed form of DNA; RMSD –root mean square deviation; SC DNA—supercoiled form of DNA; SDS—sodium dodecyl sulphate; STR—Streptomycin; Trp (Boc)—Boc-L-tryptophan; TRIS HCl—Tris (hydroxymethyl) aminomethane hydrochloride.
